# Comparison of Survival Rates, Tumor Stages, and Localization in between Obese and Nonobese Patients with Gastric Cancer

**DOI:** 10.1155/2016/9382750

**Published:** 2016-06-21

**Authors:** Hakan Kocoglu, Hakan Dogan, Basak Oguz, Sibel Ocak Serin, Yildiz Okuturlar, Meral Gunaldi, Betul Erismis, Bahar Ozdemir, Deniz Tural, Mehmet Hursitoglu, Ozlem Harmankaya, Abdulbaki Kumbasar

**Affiliations:** ^1^Department of Internal Medicine, Bakirkoy Dr. Sadi Konuk Education and Research Hospital, 34147 Istanbul, Turkey; ^2^Department of Internal Medicine, Izmir Bozyaka Education and Research Hospital, 35170 Izmir, Turkey; ^3^Department of Internal Medicine, Istanbul Bilim University, Florence Nightingale Hospital, 34394 Istanbul, Turkey; ^4^Department of Internal Medicine, Umraniye Education and Research Hospital, 34766 Istanbul, Turkey; ^5^Department of Medical Oncology, Bakirkoy Dr. Sadi Konuk Education and Research Hospital, 34147 Istanbul, Turkey

## Abstract

*Purpose.* In this study we tried to determine the association between body-mass index (BMI), survival rate, and the stage of tumor at the time of diagnosis in patients with gastric cancer.* Methods.* A total of 270 gastric cancer patients' hospital records were retrospectively evaluated. Patients were grouped according to their BMI at the time of tumor diagnosis. Tumor stages at admission were compared according to their BMI values.* Results.* There were no differences in OS among BMI subgroups (*p* = 0.230). The percent of patients with stage III tumor was significantly higher in nonobese while the percent of stage IV tumor was surprisingly higher in obese patients (*p* was 0.011 and 0.004, resp.). Percent of patients who did not have any surgical intervention was significantly lower in overweight and obese patients than normal and/or underweight patients.* Conclusions.* At the time of diagnosis, obese patients had significantly higher percent of stage IV tumor than nonobese patients. Despite of that, there were no differences in survival rates among BMI subgroups. Our study results are consistent with “obesity paradox” in gastric cancer patients. We also did not find any relationship between BMI and localization of gastric tumor.

## 1. Introduction

The overall gastric cancer incidence and mortality rates have been decreasing worldwide, but despite this decline, gastric cancer remains the fourth most common cancer and the second leading cause of cancer related mortality and also it is predicted that the number of gastric cancer cases globally will increase until 2050 [1–3]. Although the incidence is declining due to improved nutrition, food preservation, better prevention, earlier diagnosis, and treatment, the disease still carries a poor prognosis. Gastric cancer is often diagnosed at an advanced stage. The cornerstone of therapy is surgical resection with adjuvant chemotherapy or chemoradiation in appropriate cases. Such an approach has led to improved survival [4–6].

Obesity is a worldwide epidemic and the prevalence of obesity, as measured by body mass index (BMI, defined as a BMI ≥ 30 kg/m^2^), has grown markedly over the past 2 decades. If recent trends continue, by 2030 up to 57.8% of the world's adult population (3.3 billion people) could be either overweight or obese. Moreover, compared with developed regions of the world, developing regions are projected to have a much larger proportional increase in the number of overweight and obese individuals between 2005 and 2030 [7]. A narrative review of the WCRF/AICR (2007) report has linked obesity with an increased risk for several cancers including cancers of the gastric, colon, breast, esophagus (adenocarcinoma), endometrium, and kidney as well as additional sites possibly [8].

Even though mechanisms underlying this association are completely unknown, it is well known that obesity increases the risk of gastric cancer and may affect its development and progression. Meta-analyses demonstrated that the body mass index is closely correlated with the risk of gastric cancer [9], particularly cardia gastric cancer [10].

As the world population has been becoming obese, gastric cancer will become an increasing clinical challenge. To date, while it is well known that increased BMI is associated with increased risk of gastric cancer, no information is available in regard to association between BMI and survival in gastric cancer patients. Also whether or not the stage of gastric cancer differs according to the patients BMI at the time of its diagnosis is not known.

## 2. Materials and Methods

This study was approved by institutional ethical committee of the Bakirkoy Dr. Sadi Konuk Education and Research Hospital, which complies with Helsinki Declaration. The study included follow-up records of 270 gastric cancer patients who were treated at oncology clinic of our hospital (Istanbul, Turkey). Patients who had the diagnosis code of gastric cancer (according to the International Classification of Diseases for Oncology) were enrolled in this study [11]. The cases in whom adenocarcinoma diagnosis was confirmed histologically and whom follow-up and survival data were complete were included in final analysis. Patients whose tumors were identified as other than adenocarcinoma were excluded from study. The patients' characteristics with respect to age, sex, BMI (at the time of diagnosis), TNM stage, and tumor localization (according to the American Joint Committee on Cancer, AJCC 7th edition) [12], Eastern Cooperative Oncology Group (ECOG) performance status, comorbid illnesses, anemia status, histopathological type of tumor, metastasis status of cancer, surgical intervention status, and received chemotherapy regimen were recorded for data analyses. Patients were divided into subgroups according to their BMI values (underweight ≤ 18.5; normal weight = 18.5–24.9; overweight = 25–29.9; obesity ≥ 30 kg/m^2^). According to patients' data, 5-year overall survival (OS) of all groups was calculated. OS was calculated from the time of diagnosis until the date of death or the date the patient was last known to be alive. The mortality rate was calculated as the number of deaths before June 30, 2015, divided by the number of person-years at risk for death.

*Statistical Analysis*. Statistical analyses were made using NCSS (Number Cruncher Statistical System) 2007 (NCSS, LLC Kaysville, Utah, USA). During the evaluation of study variables, descriptive statistical methods (mean, standard error, median, rate, and ratio) were used. Data were analyzed using Chi-square test, Student's *t*-test, Pearson Chi-square test, and Fisher Freeman Halton test as appropriate. Cancer specific survival rates were estimated by the Kaplan-Meier method and cancer relative survival was calculated from the date of diagnosis until death. The statistical level of significance was defined as *p* < 0.05.

## 3. Results

The hospital-based registry included a total number of 270 gastric cancer patients which consisted of 195 male (72.2%) and 75 female (27.8%) subjects (*p* = 0.0001). Mean age was 59.4 ± 11.9 years and mean BMI was 23.15 ± 4.2 kg/m^2^. Nineteen (7%) patients were stage I; 25 (9.3%) patients were stage II; 109 (40.4%) patients were stage III; and 117 (43.3%) patients were stage IV (Table 1). Also there was no difference regarding age and gender among BMI subgroups (*p* > 0.05 for all).

**Figure 1:**
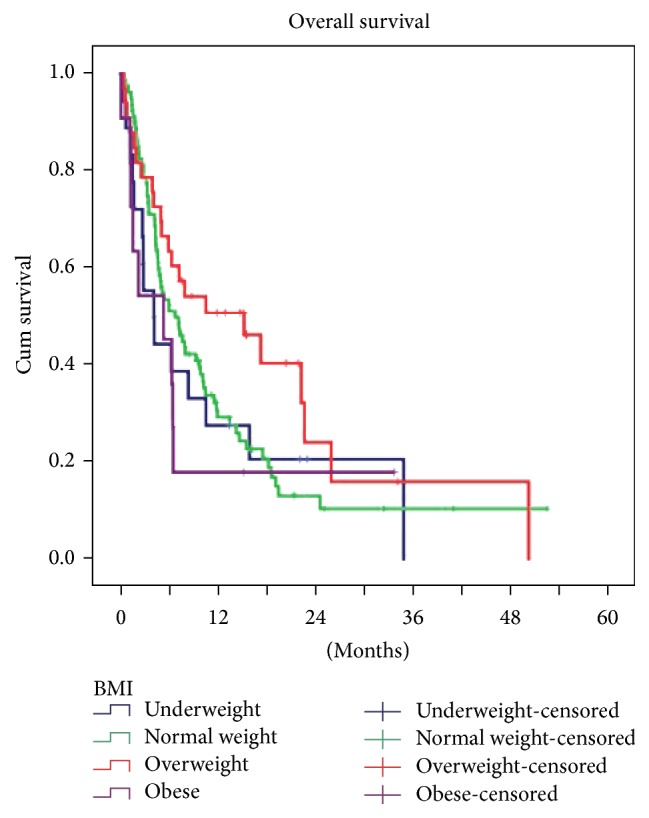
Relation between body mass index and overall survival in patients with gastric cancer (Kaplan-Meier curves).

Mean mortality rate was 79.5% and mean OS time was 26.6 months. Mortality rates of male and female patients were 83.5% and 69.6%, respectively, and there was no difference in mortality rates among genders (*p* = 0.225). Percent of underweight (<18.5 kg/m^2^), normal weight (18.5–24.9 kg/m^2^), overweight (25–29.9 kg/m^2^), and obese (≥30 kg/m^2^) patients and mean OS times were, respectively, as follows: 12.6% (22.2 months), 56.3% (25.4 months), 23.3% (35.4 months), and 7.8% (18.1 months). There were no differences in OS among BMI subgroups (*p* = 0.230) (Figure 1). As expected there was statistical significant difference in survival rates among tumor stages (*p* = 0.001) (Table 1).

When we compared BMI and tumor stages, the percent of patients with stage III tumor was significantly lower in patients with obesity than in those without obesity (*p* = 0.011). On the other hand, the percent of patients with stage IV tumor was significantly higher in patients with obesity than in those without obesity (*p* = 0.004). But the percent of patients with stages I and II did not differ according to their body weights. Tumor localization did not differ according to BMI (Table 2).

When we compared BMI and surgery status and type, it was seen that the percent of patients who did not have any surgical intervention was significantly lower in overweight and obese patients than underweight and normal patients (*p* = 0.012) (Table 3).

## 4. Discussion

BMI has become a widely used variable in clinical practice, classifying specific sets of comorbidities and differential clinicopathological characteristics [13]. In essence, a low BMI could be accompanied by low albumin and hemoglobin levels and this could become obvious in cancer patients because of the energy disturbance resulting from cachexia [14].

BMI is associated with several types of cancer, including gastric cancer [9]. A previous study has shown that diet-induced obesity potentiates the growth of gastric cancer in mice [15]. Also a meta-analysis has shown that increased BMI was positively associated with the risk of gastric cardia cancer but not with gastric noncardia cancer [10].

Despite all these facts, the relation between survival rate and BMI has not been fully clarified yet. Also whether or not obese gastric cancer patients have advanced stage at the time of diagnosis is not known. Thus in this study we aimed to determine whether or not there is a difference in survival rates or tumor stages at the time of diagnosis between obese and nonobese patients. When we compared BMI and tumor stages, percent of patients with stage IV tumor was significantly higher in patients with obesity than in those without obesity. This finding could be caused by several factors. In a murine model study, obesity has been shown to be provocative and early metastatic feature. Another important point to mention is that unexpected weight loss (due to malignancy) may be ignored or unnoticed in overweight patients because weight loss is a desired thing in such patients. Furthermore, performing invasive procedures (e.g., endoscopic examinations) is somewhat difficult in obese patients which may lead to a delay in decision of performing such investigations and eventually a delay in diagnosis.

Our study results also showed that although obese patients had advanced stages of cancers, they had similar survival outcomes to nonobese persons. Nutritional status, which is expected to be better in obese patients, is one of the key determinants of survival in patients with gastric cancer [16]. But whether or not our results are influenced from the fact that the percent of patients who did not have any surgical intervention was significantly lower in overweight and obese patients than underweight and normal patients is not clear. There are controversial studies in regard to the impact of BMI on the long-term outcomes of gastric cancer patients who underwent gastrectomy. Chen et al. have shown that low BMI may be associated with poor prognosis among stages III-IV gastric cancer patients, exhibiting a paradoxically “superior” survival outcome compared with normal-BMI patients [17]. A meta-analysis by Wu et al. [18] demonstrated that gastric cancer patients with a BMI ≥25* *kg/m^2^ correlated with poor long-term survival. However, a study from Asia found no significant difference in 5-year OS among patients with BMI <25, 25–30, and >30* *kg/m^2^ [19]. In another study, Ejaz et al. [20] found that patients with BMI <18.5* *kg/m^2^ had a significantly decreased OS after gastrectomy while OS did not differ among normal-BMI and high-BMI patients. More recently, Wong et al. [21] stated that overall survival and disease-free survival were significantly associated with increased BMI. However, BMI did not associate with OS in multivariate analysis. Although obese subjects are more prone to have gastric cancer when compared to nonobese subjects, overweight and mild obesity are protective against mortality and this interesting paradox has been referred to as the “obesity paradox” [17, 22, 23]. This paradox might also have contributed to these study results.

Several studies have been published on the relationship between BMI and localization of gastric tumor (cardia versus noncardia) and the study results are controversial. Some studies which include very large study populations showed no relation between BMI and gastric cardia adenocarcinoma [24, 25], whereas the most of the studies showed elevated risk of gastric cardia adenocarcinoma in obese patients [26]. Because the gastric cardia is an anatomically small region, definitional issues, not surprisingly, exist. In 1990s Siewert classification system for gastroesophageal junction tumors has been in common use throughout the world (also sixth edition of AJCC staging system corresponds to Siewert types 2 and 3). But in the seventh edition of AJCC staging system (2010) gastroesophageal junction tumors were redefined and Siewert types 2 and 3 tumors are considered to be esophageal cancers. Thus, the definition of cardia, study design, and number of cases could be associated with these controversial results. In contrast to most of the previous studies which have used Siewert classification system/sixth edition of AJCC staging system, seventh edition of AJCC staging system has been used in our study.

## 5. Conclusion

At the time of diagnosis, the percent of patients with stage IV tumor was significantly higher in obese patients than nonobese patients. Despite having advanced stages of cancer in obese patients, overall survival did not differ according to BMI. Our study results are consistent with previous studies of which findings confirmed the “obesity paradox” in gastric cancer patients. We also did not show any relationship between BMI and localization of gastric tumor. Further studies are needed to clarify these points.

## Figures and Tables

**Table 1:** Clinicopathological features of all patients with respect to gender, BMI (body mass index), and stage of cancer.

			Mean OS (month)	Mortality rate	*p*
*Gender*	*n*	*p*			
Female	75	0.0001	34	69.6%	0.225
Male	195		23.9	83.5%	

*BMI*	*n*	*%*			
Underweight	34	12.6	22.2	83.3%	0.23
Normal	152	56.3	25.4	81.3%	
Overweight	63	23.3	35.5	66.7%	
Obese	21	7.8	18.1	81.8%	

*Stage*	*n*	*%*			
I	19	7	77	42.9%	**0.001**
II	25	9.3	36.8	66.7%	
III	109	40.4	35.2	68.7%	
IV	117	43.3	10.8	93.7%	

**Table 2:** Comparison between tumor stage, tumor localization, and body mass index (BMI) in gastric cancer patients.

		BMI				*p*
		Underweight	Normal	Overweight	Obese	
Stage						
I	*n*	2	10	5	2	0.126
	%	5.9	6.6	7.9	9.5	
II	*n*	3	14	5	3	0.357
	%	8.8	9.2	7.9	14.3	
III	*n*	14	67	26	2	**0.011**
	%	41.2	44.1	41.3	9.5	
IV	*n*	15	61	27	14	**0.004**
	%	44.1	40.1	42.9	66.7	

Tumor localization						
Cardia	*n*	2	17	8	2	0.226
	%	5.89	11.19	12.70	9.53	
Noncardia	*n*	32	135	55	19	0.458
	%	94.11	88.81	87.30	90.47	

**Table 3:** Comparison between type of gastric surgery and body mass index in gastric cancer patients.

	BMI								*p*
	Underweight		Normal		Overweight		Obese		
	%	*n*	%	*n*	%	*n*	%	*n*	
Surgery									
None	34.8	12	31.1	47	6.0	4	9.6	2	**0.012**
Subtotal gastrectomy	26.1	9	20.9	32	31.3	20	33.8	7	0.658
Total gastrectomy	39.1	13	48.0	73	62.7	39	56.5	12	0.216
